# Using the SLIPTA checklist to assess laboratory readiness for Joint Commission International accreditation

**DOI:** 10.4102/ajlm.v12i1.2044

**Published:** 2023-03-15

**Authors:** Abdul K. El Karaaoui, Nada Assaf

**Affiliations:** 1 Department of Pathology and Laboratory Medicine, Faculty of Medicine, American University of Beirut, Beirut, Lebanon

**Keywords:** standard culture, Joint Commission International, laboratory accreditation, low- and middle-income countries

## Abstract

**Background:**

The Stepwise Laboratory Improvement Process Towards Accreditation (SLIPTA) helps prepare laboratories in low- and middle-income countries to achieve international accreditation aligned with the ISO 15189:2012 standards. Accreditation by the Joint Commission International (JCI) is among the highest sought by hospitals worldwide. While the readiness of laboratories with a five-star SLIPTA score to undergo ISO 15189:2012 accreditation was recently assessed, the compliance of the SLIPTA checklist with JCI is still unknown.

**Objective:**

The study evaluated the SLIPTA checklist’s utility in assessing laboratories to meet the JCI standards.

**Methods:**

We conducted a detailed gap analysis between SLIPTA and JCI laboratory standards from January 2021 to January 2022. We cross-matched the JCI standard requirements to SLIPTA clauses and categorised each standard into ‘met’, ‘partially met’, and ‘not met’. We highlighted similarities, discrepancies, and improvement areas.

**Results:**

A total of 109 JCI standards were included. The SLIPTA checklist completely met 61 standards, partially met four, but did not meet 44. The unmet JCI standards focused on the quality planning, control, and improvement sections. Healthcare organisation management and quality control processes, including selecting an accredited reference laboratory, collecting quality management data, creating of post-analytical policies and procedures, and validating monitoring systems, constitute the basis of this preparation.

**Conclusion:**

The SLIPTA checklist covers major quality management system elements of the JCI standards for laboratories. However, some components should be addressed to assure readiness for JCI accreditation.

**What this study adds:**

This study identified additional areas not covered by the SLIPTA checklist that are required for JCI accreditation.

## Introduction

Medical laboratories play a pivotal role in maintaining the healthcare chain in a nation. Laboratory services are crucial for disease prevention, diagnosis, surveillance, and treatment.^1,2^ In developed countries, up to 70% of clinical decisions depend on laboratory test results.^3,4^ As such, maintaining quality laboratory services is essential for the efficient functioning of the healthcare system.

Accreditation is an external quality review that evaluates an organisation’s conformity with a predefined set of standards to receive formal recognition of compliance by an authorised external body.^5^ As a primary goal, laboratory accreditation aims to improve patient safety and the standard of care.^6^ In addition, accredited laboratories show superior test reliability, better operational efficiency, and higher competence.^7^

With increased outbreaks of diseases such as HIV, tuberculosis, and the recently emerging coronavirus disease 19 in low- and middle-income countries (LMICs), there is increased attention on strengthening healthcare systems in limited-resource areas.^8^

In 2009, the World Health Organization Regional Office for Africa, in collaboration with the African Society for Laboratory Medicine, the United States Center for Disease Control and Prevention and African countries consorted on the creation of a Stepwise Laboratory Improvement Process Towards Accreditation (SLIPTA) to implement quality management systems (QMS) and organise quality improvement processes in LMIC laboratories.^9^ The SLIPTA programme consists of a checklist questionnaire linked to a five-star recognition depending on the level of compliance ranging from ≤ 55% (0 stars) to ≥ 95% (5 stars).^8,9^ It is an interim recognition, and laboratories that achieve a SLIPTA five-star rating are encouraged to apply for international accreditations such as ISO 15189:2012 and Joint Commission International (JCI).^8^

Founded in 1951, JCI is today the largest international accreditation body for hospitals.^11^ The JCI laboratory accreditation standards cover all laboratory needs for continual improvement within a quality management environment.^11^ It also addresses essential laboratory managerial and clinical functions while focusing on patient safety.^11^ Because JCI is a hospital accreditation scheme, laboratory JCI clauses are clinically oriented. Therefore, it is complementary to ISO 15189 standards, which are process based. As an increasing number of hospitals worldwide are seeking JCI accreditation, laboratories (including LMIC laboratories) might undergo on-site evaluation by the JCI during their hospital accreditation inspection.

The suitability of the latest version of SLIPTA to guide laboratories towards an ISO 15189:2012 accreditation was recently assessed by Datema et al.^8^ However, the readiness of SLIPTA five-star laboratories to undergo JCI accreditation is yet undetermined. The aim of this study was to evaluate the SLIPTA checklist’s usefulness in assessing laboratories to meet JCI standards.

## Methods

### Ethical considerations

The local institutional review board (Human Research Protection Program at the American University of Beirut) deemed the study exempt from review. This study did not involve human or non-human participants and did not require participant consent or data protections.

### Study design

This study was conducted at the College of American Pathologists accredited Department of Pathology and Laboratory Medicine at the American University of Beirut Medical Center, Lebanon. The American University of Beirut Medical Center is a JCI-accredited tertiary healthcare facility. The assessors (the manuscript’s two authors) were selected based on their experience in the different accreditation processes and their certification as auditors for select certification bodies. The latest versions of SLIPTA (version 2:2015) and JCI standards for laboratories (3rd edition; January 2017) were retrieved from the World Health Organization Regional Office for Africa and JCI websites^9,10,11^.

### Data analysis

After thoroughly reviewing the JCI and SLIPTA standards, the authors performed a gap assessment separately between January 2021 and January 2022. First, each JCI clause was compared to its counterpart in the SLIPTA checklist and categorised as a ‘met’, ‘partially met’, or ‘not met’. ‘Met’ JCI clauses were addressed and totally covered by the SLIPTA checklist, ‘partially met’ standards were addressed but not all elements were covered by the checklist, while ‘not met’ standards were not addressed. Percentages were calculated using a spreadsheet, Microsoft Excel (Microsoft, Redmond, Washington, United States). We then compared each author’s results, discussed areas of discrepancies, and issued a unified consensual analysis. The JCI standards were divided into the three categories of the Juran Trilogy model (quality planning, quality control and quality improvement) as similarly done by Juran.^12^ The results of our scoring system were used to determine the categories with the highest percentage of gaps.

The SLIPTA comprises 12 sections, each corresponding to a single QMS element, which can be grouped into three categories: ‘quality planning’, ‘quality control’ and ‘quality improvement’, according to the Juran Trilogy model.^12^

The JCI booklet comprises three general divisions, ‘accreditation participation requirements’, ‘patient-centred standards’, and ‘healthcare organisation management standards’, subdivided into seven sections.

Of the 152 JCI standards, 109 were reviewed and compared to their counterparts in the SLIPTA checklist. Forty-three standards in Sections 1 and 7 of JCI were excluded from this review. Section 1 targets accreditation participation requirements. This section is specific to JCI and includes general principles such as the timely submission of accurate and complete data to the JCI committee. Section 1, including its 12 clauses, was excluded from the gap analysis due to lacking a counterpart. Section 7 includes standards covering speciality quality control process (QCP) (QCP. 2 – QCP. 10.1 and QCP. 26 – QCP. 27.5: 31 clauses), which details requirements for single laboratory speciality sections. Because SLIPTA views hospital laboratories as a single unit, it lacks speciality-specific questions, and the corresponding standards (20%; 31/152) were excluded from the analysis.

## Results

The SLIPTA V2:2015 addresses 60% of the 109 JCI standards reviewed (56%; 61/109 wholly met and 4%; 4/109 partially met) but does not meet 40% (44/109) (Table 1). Among ‘partially met’ standards, 75% (3/4 standards) belong to the quality planning category and 25% (1/4) to quality improvement (Table 2). Among the ‘not met’ JCI clauses, 11% (5/44) are quality planning clauses, 82% belong to quality control (36/44) and 7% (3/44) to quality improvement.

**TABLE 1:** Gap analysis of Joint Commission International and Stepwise Laboratory Improvement Process Towards Accreditation, January 2021 – January 2022.

JCI section	Topic	JCI standard	Status in SLIPTA	Corresponding standard in SLIPTA
JCI section 2: Patient safety Goals	Accurate patient identification process	IPSG.1	Met	Section 8.1 (Process control) Section 8.2 (Process control)
	Effective communication and reporting of critical results	IPSG.2	Met	Section 1.5 (Communication internal and external) Section 1.5 (Reporting and release of results)
	Evidence-based hand hygiene to decrease the risk of infections.	IPSG.5	Met	Section 3.6d (Organisation and personnel) Section 12.15c (Facilities and biosafety)
JCI section 3: Healthcare organisation management	Identifying leadership and its role in determining the laboratory’s mission and vision.	GLD.1	Partially met	Section 3.2
	Qualification required for the laboratory director	GLD.1.1	Met	Section 3.3
	Professional qualification to address patients’ and clients’ needs	GLD.1.2	Partially met	Section 4.1 Section 4.5
	Service and resource planning	GLD.2	Not met	–
		GLD.2.1	Met	Section 2.2
	Selecting contract and reference laboratory services	GLD.2.2	Met	Section 1.5 (Examination by referral laboratories and consultants) Section 3.3f Section 8.6
		GLD.2.2.1	Not met	–
		GLD.2.2.2	Met	Section 1.5 (Examination by referral laboratories and consultants) Section 8.6
	Blood bank and transfusion services	GLD.2.3	Not met	–
	Point of care services	GLD.2.4	Not met	–
	Communication and coordination	GLD.3	Met	Section 1.5 (Communication internal and external)
		GLD.3.1	Met	Section 1.5 (Service agreements)
		GLD.3.2	Met	Section 1.5 (Communication internal and external)
	Planning and coordination of the quality management and improvement programme design of new processes	GLD.4 GLD.4.1 GLD.4.2 GLD.4.3 GLD.4.4	Met	Section 1.5 (Continual improvement) Section 3.3 (d) Section 3.4 Section 11.2
	Data collection for quality measurement	GLD.4.5	Not met	–
		GLD.4.5.1	Not met	–
		GLD.4.5.2	Not met	–
	Analysis of measurement data	GLD.5	Met	Section 6.1c
		GLD.6	Met	Section 6.1
		GLD.7	Met	Section 1.5 (Validation and Verification of examination procedures / Equipment) Section 6.1d-e Section 11.1
	Gaining and sustaining improvement	GLD.8	Met	Section 1.5 (Continual improvement) Section 2.2 Section 11.2 Section 11.3
	Safety risks and adverse events	GLD.9	Met	Section 1.5 (Laboratory safety manual) Section 12.4 Section 12.9 Section 12.14 Section 12.15
	Quality management and improvement programme review	GLD.10	Met	Section 2.2
	Laboratory leadership for resource decisions	GLD.11	Met	Section 2.2
	Culture of safety	GLD.12	Met	Section 3.3g
		GLD.12.1	Partially met	Section 12.14
JCI section 4: Management of information	Management and implementation of documents	MOI.1	Met	Section 1.2
		MOI.1.1	Met	Section 1.2
	Preanalytical policies and procedures	MOI.2	Met	Section 1.5 (Pre-examination process) Section 8.4
		MOI.2.1	Met	Section 1.5 (Pre-examination process) Section 8.4
		MOI.2.2	Met	Section 1.5
	Analytical policies and procedures	MOI.3	Met	Section 1.5 (Documentation of examination procedures) Section 8.7
	Post-analytical policies and procedures	MOI.4	Not met	
		MOI.4.1	Met	Section 11.4
		MOI.5	Met	Section 1.5 (Control of records) Section 1.10 Section 9.5 Section 12.6
JCI section 5: Staff qualifications and education	Human resources	SQE.1	Met	Section 3.3c
	Staff qualifications	SQE.1.1 SQE.1.1.1 SQE.1.2	Met	Section 3.5
	Staff orientation and education	SQE.2	Met	Section 3.3i Section 3.6
	Competence assessment and performance evaluation	SQE.3	Met	Section 3.7
		SQE.3.1	Met	Section 3.5
		SQE.4	Met	Section 3.6d
JCI section 6: Facility management and safety	Infrastructure – Basic facilities	FMS.1	Met	Section 1.5 (Accommodation and environmental conditions)
	Laboratory space and resources	FMS.2	Met	Section 12.1
	Proper and sufficient storage area	FMS.2.1	Met	Section 12.6
	Protected records and patient data	FMS.2.2	Met	Section 1.5 (Control of records)
	Utilities management	FMS.3	Met	Section 5.1 Section 5.3
		FMS.3.1	Met	Section 5.10 Section 5.11
		FMS.3.2	Met	Section 5.14
	Laboratory equipment and other materials are adequate and available	FMS.4.	Met	Section 1.5 (Laboratory equipment) Section 5.1
	Maintenance, inspection, and testing laboratory equipment with documentation of records	FMS.4.1	Met	Section 1.5 (Laboratory equipment) Section 5.3
	Record of analytical instruments and equipment used by laboratory	FMS.4.1.1	Met	Section 1.5 (Laboratory equipment) Section 5.5
	Defined processes for validating and maintaining computer software and information	FMS.4.2	Met	Section 9.9 Section 9.10
	Documentation of required information for reagents	FMS.4.4	Met	Section 7.4 Section 8.8
	Safety and security	FMS.5	Met	Section 12.9 Section 12.14
	Hazardous materials and waste handling and disposal	FMS.6	Met	Section 12.9c Section 12.10 Section 12.11
		FMS.6.1	Met	Section 12.11
	A process to reduce risk of infection	FMS.6.2	Partially met	Section 12.7
	Safe handling and use of radioactive substances	FMS.6.3	Not met	–
	Work environment – Laboratory fire safety	FMS.7	Met	Section 12.9h Section 12.13
		FMS.7.1	Met	Section 12.12d
JCI section 7: Quality control processes	Established quality control processes for each test method	QCP.1	Met	Section 1.2
	External graded interlaboratory comparison testing, or proficiency testing	QCP.1.1	Met	Section 8.14
		QCP.1.1.1	Met	Section 8.14b
	Verification of the accuracy and reliability of test results	QCP.1.1.2	Met	Section 8.10
	Comparability of methods used for same test	QCP.1.2	Met	Section 8.11
	Validation of new instruments	QCP.1.3	Met	Section 5.3
	Validation of monitoring systems used for quality control	QCP.1.4	Not met	–
	Calibration	QCP.1.5	Met	Section 1.5 (Calibration of equipment) Section 5.9
	Process for a coordinated review of patient results, quality control results, and instrument function checks	QCP.1.6	Met	Section 2.1
	Corrective actions for deficiencies	QCP.1.7	Met	Section 10.3 Section 10.4 Section 10.5
	Specialty quality control	QCP.11 to QCP.25.5	Not met	–

JCI, Joint Commission International; SLIPTA, Stepwise Laboratory Improvement Process Towards Accreditation. IPSG, International Patient Safety Goals; GLD, governance, leadership and direction; FMS, facility management and safety; SQE, staff qualification and education; QCP, quality control processes; MOI, management of information.

**TABLE 2:** Distribution of non-conformities between Joint Commission International clauses and the Stepwise Laboratory Quality Improvement Process Towards Accreditation checklist based on the categories of the Juran Trilogy, January 2021 – January 2022.

Category of the Juran Trilogy model	Quality planning	Quality control	Quality improvement
Met JCI standard	GLD 1.1 GLD 2.1 GLD 2.2 to 2.2.2 GLD 3 GLD 11 GLD 12 to 12.1 FMS 1 to 4 FMS 5 to 6.1 FMS 7 to 7.1 - -	GLD 5 GLD 6 GLD 7 GLD 9 MOI 1 to 3 MOI 4.1 MOI 5 SQE 1 to 4 FMS 4.1 to 4.4 QCP 1 to 1.3 QCP 1.5 to 1.7	GLD 4 to 4.4 GLD 8 GLD 10 - - - - - - - -
Partially met JCI standard	GLD 1 GLD 1.2 FMS 6.2	- - -	GLD 12.1 - -
Unmet JCI standard	GLD 2 GLD 2.2.1 GLD 2.3 GLD 2.4 FMS 6.3	MOI 4 QCP 1.4 QCP.11 to QCP.25.5 - -	GLD 4.5 GLD 4.5.1 GLD 4.5.2 - -

JCI, Joint Commission International; SLIPTA, Stepwise Laboratory Improvement Process Towards Accreditation; QMP, quality control process.

Gaps were noted in standards related to the organisation’s vision and mission statement, the accreditation of referral laboratories, ongoing communication with stakeholders, and quality monitoring. In terms of quality control, the validation of monitoring systems before implementation was also missing in the SLIPTA checklist.

### SLIPTA checklist coverage of Joint Commission International standards

#### Joint Commission International Section 2: Patient safety goals

This section focuses on patient safety goals and includes correct patient identification, effective communication of critical results, and hand hygiene standards. Section 8 of the SLIPTA (process control) addresses all the requirements for preanalytical quality checks, including patient identification, sample collection and reception. The first section of the SLIPTA covers effective reporting of critical results, including adequate documentation and record keeping, while the facilities and biosafety section cover the hand hygiene standards. In addition, the SLIPTA standards require proper staff training on hand hygiene practices (section 3.6 d).

#### Joint Commission International Section 3: Healthcare organisation management

The JCI section 3 consists of healthcare organisation management standards, for which the initial step is the design of a leadership organisational chart and the determination of the laboratory’s mission and vision. The SLIPTA checklist enquires about the presence of an organisational chart and a narrative description (section 3.2) of the different internal and external relationships within the laboratory. However, no points are assigned to developing a vision and mission statement. The following organisation management requirement is the appointment of a qualified laboratory manager, a clause fulfilled in the SLIPTA checklist. Despite the presence of a section in the SLIPTA checklist addressing client management and customer service, there is no requirement for a qualified individual to oversee these procedures. In the JCI, management is also responsible for service organisation and continuous communication with stakeholders; however, this is uncovered by the SLIPTA checklist. Resource planning is addressed in section 2.2 of the SLIPTA checklist as part of the output review by leadership.

The following section in JCI regulates the contracts and services with reference laboratories, namely the presence of standard operating procedures to define the process for selecting and approving contracts and services by the leadership (governance, leadership and direction [GLD] [GLD.2.2]), the need for proof of accreditation or certification (GLD.2.2.1), and performance monitoring (GLD.2.2.2). While the SLIPTA sets detailed requirements for the choice of referral laboratories and their continuous monitoring, along with the responsibility of the laboratory director in these tasks, it does not require checking the accreditation status of referral laboratories.

The SLIPTA checklist views the laboratory as a single unit rather than different laboratory speciality sections. Thus, unlike the JCI, it does not address standards for blood bank (GLD.2.3) and point-of-care services (GLD.2.4). Nevertheless, the SLIPTA standard 3.3, section 3, requires a laboratory director with adequate competency to supervise all laboratory workstations, which by extension includes blood bank and point-of-care services, as required by the JCI GLD.2.3 and GLD2.4.

In the JCI, GLD.3, 3.1 and 3.2 regulate effective inter- and intra-laboratory communication and efficient determination of clients’ needs; these are also detailed in SLIPTA.

The next JCI section defines the different aspects of planning and coordination of a quality management programme, including the responsibilities of the leadership in developing the programme (GLD.4) and implementing activities (GLD.4.3), along with the required criteria for a well-functioning programme (GLD.4.1) and its monitoring by a qualified individual (GLD.4.2). The SLIPTA checklist conforms to the JCI requirements for quality management design. However, it does not completely cover quality management monitoring. For example, the JCI quality management monitoring the leadership to determining processes to be measured (GLD.4.5) and standards required to identify critical measures for clinical (GLD.4.5.1) and managerial (GLD4.5.2) structures, all of which SLIPTA does not address. In addition, the analysis of measurement data requires skilled individuals (GLD.5), an internal process to validate data (GLD.6) and an appropriate investigation of off-target trends (GLD.7). These criteria are met in the SLIPTA checklist sections 1.5, 6.1 and 11.1 that determine the need for effective auditing, including personnel, procedures, and trend verification tools.

The Joint Commission InternationalGLD.8 to 12.1 state principles for well-functioning safety and quality insurance programmes, including, but not limited to, the presence of an ongoing programme (GLD.9), and the responsibility of the leaders to create, implement (GLD.8), provide resources (GLD.11) and monitor ongoing programmes (GLD.10) while maintaining a safety culture throughout the laboratory (GLD.12 and GLD.12.1). Laboratories with a five-star SLIPTA accreditation are considered compliant with the safety and quality insurance programmes set by JCI.

#### Joint Commission International Section 4: Information management

Section 4 of the JCI focuses on information management. It states the need for the presence of written policies and procedures addressing all testing phases (preanalytical: [management of information {MOI}] MOI.1, 2, 2.1, 2.2; analytical: MOI.3; post-analytical: MOI.4) and storage (MOI.5), as well as their implementation (MOI.1.1). It also requires the definition of a turnaround time (MOI.4.1). The SLIPTA checklist requires the presence of written policies and procedures (section 1.2) and their implementation (section 1.2). Therefore, SLIPTA meets all standards for all testing phases (preanalytical: sections 1.5, 8.4; analytical: sections 1.5, 8.7; post-analytical: section 1.5), including setting turnaround time and storage requirements for records and specimens.

#### Joint Commission International Section 5: Staff qualifications and education

Section 5 standards are related to staff qualification and education. The first standard ([staff qualification and education {SQE}] SQE.1) mentions the role of laboratory leaders in defining the education and skills required for staff members, which is met by SLIPTA section 3.3 on the role of laboratory directors.

Section 3 of SLIPTA complies with the second standard in JCI Section 5, which discusses the training and experience required for supervisors and other leaders, licensure and registration of pathologists, and the need for defined responsibilities in the job descriptions.

The next JCI standard focuses on staff orientation, ongoing education, and yearly competency assessment needed to maintain and improve staff skills and knowledge (SQE.2, 2.1 and 3); the SLIPTA sections 3.3, 3.6, and 3.7 meet these requirements. Also, the SLIPTA section 3.5. mentions the JCI standard SQE.3.1 (maintenance of documented personnel information for every staff member) requirements. Finally, the last standard in this section requires the laboratory to provide health services to all staff (SQE.4) and is met by the SLIPTA checklist.

#### Joint Commission International Section 6: Facility management

Laboratory facility management constitutes the focus of Section 6. The first standard targets the responsibilities of laboratory leaders in providing the basic facilities and their facility’s compliance with laws and regulations. This requirement is met by SLIPTA section 1.5 on accommodation and environmental conditions.

The next standards state the requirement for sufficient storage space ([facility management and safety {FMS}] FMS.2.1) and proper record keeping (FMS.2.2). Resources for this goal should be provided by the laboratory leadership (FMS.2). Storage requirements, record keeping, and leadership roles are covered by the SLIPTA sections 12.6, 1.5 (records control and sections) and 12.1.

The JCIstandards FMS.3 to 4.1.1 mention the different aspects for efficient and effective operation of all utility systems and equipment. These aspects include inspection, testing of critical operating components, maintenance, and availability of emergency backup for critical utilities. Interestingly, SLIPTA section 1.5 on laboratory equipment and section 5 on equipment give a more detailed description of proper equipment protocol due to the question format checklist.

The JCI standard FMS.4.2 on the requirement for a defined process for validating and maintaining computer software and laboratory information is met by SLIPTA sections 9.9 and 9.10.

The standards for periodic evaluation, labelling and documentation of reagents are met in SLIPTA sections 7.4 (inventory control) and 8.8 (reagents acceptance testing).

Finally, section 12 in SLIPTA (12.8–12.13) covers all safety aspects required by the last JCI section 6 standard on safety (FMS.5), including the management of hazardous materials, waste (FMS.6 to 6.3), and fire (FMS.7 and 7.1).

#### Joint Commission International Section 7: Quality control

The last JCI section deals with quality control (QC) standards for all testing areas. It includes establishing QC processes for the different test methods, external and internal QC, verification of results accuracy, and methods comparison. It also targets new instrument validation and instrument monitoring systems implementation. The QC processes are addressed through several SLIPTA sections with high compliance with JCI clauses. However, the SLIPTA checklist does not have questions on the validation of all monitoring systems before their implementation, as required by JCI.

## Discussion

The level of compliance of SLIPTA with JCI requirements appears to be lower than its compliance with ISO 15189, as determined by Datema et al.^8^ This observation is because the SLIPTA checklist is primarily based on the ISO 15189 and the Clinical and Laboratory Standards Institute guideline QMS01-A4 clauses in 2009.^9^

The SLIPTA checklist and JCI standards are structured differently. In addition to its checklist format, SLIPTA is more detailed and extensive than JCI, with specific instructions. The structure difference is because of their respective perspectives and objectives. The SLIPTA checklist leans towards operations rather than management and targets laboratories with newly implemented QMS. On the other hand, the JCI accreditation standards are more outcome based than process based. They focus on the role of the laboratory leadership and management structure in achieving the desired quality outcomes, resource management, QC processes, the laboratory environment and development. The JCI thus targets healthcare organisations with a particular quality background to achieve higher excellence. The JCI booklet contains general standards addressing the laboratory as a single unit (similar to the SLIPTA checklist). This approach is important to cover essential laboratory QMS elements. In addition, requirements related to specific sections within the laboratory (blood bank, haematology, chemistry) are delineated. Quality managers of laboratories undergoing JCI evaluation might benefit from ensuring that section-specific requirements are met as they constitute up to 20% of JCI standards.

Our analysis also reveals that the SLIPTA quality planning and control sections contain the highest number of gaps when compared to JCI (Table 2). As an example, the definition of the organisation’s mission and vision, which SLIPTA does not address, is crucial in the initial steps of the establishment of any QMS. Similarly, the role of leadership in service and resource planning and the implementation of culture safety should be emphasised in SLIPTA. Also, in the quality planning section, the safety programme should be improved in future versions, focusing on reducing infection risks and handling radioactive material.

Essential QC elements are missing in the SLIPTA checklist. The relationship with external (referral) laboratories should be regulated. Another important requirement for JCI is the validation of QC systems before their implementation. The post-analytical testing phase should receive special focus for the improvement of the next versions, with the appropriate policies, procedures, and controls. In terms of quality improvement, our analysis reveals a need for the improvement of the managerial decisions of improvement activities and the associated data collection for quality measurement to comply with JCI. However, the SLIPTA checklist is skewed towards quality planning and QC sections, with quality improvement having the lowest number of allocated points.^8^ Thus laboratories might focus on quality planning and QC clauses while neglecting quality improvement, which is a critical element in JCI accreditation.^8^

### Limitations

This study reviewed the SLPITA and JCI checklists; thus, limitations are due to possible human errors during the review. Each of the manuscript’s authors performed a full review independently to minimise errors.

### Conclusion

This article encourages all laboratories in LMICs seeking a QMS programme to start by implementing SLIPTA, as per the World Health Organization Regional Office for Africa recommendations. Laboratories with high SLIPTA scores seeking JCI accreditation might benefit from addressing the following elements: selecting an accredited reference laboratory, collecting data for QM, creating post-analytical policies and procedures, and validating monitoring systems used in QC. Because gap analysis is the initial step of any accreditation transition process, this analysis constitutes a useful guide for laboratory quality managers and directors in LMICs planning to undergo the JCI accreditation process as part of their healthcare organisations.
